# Exposure to third-hand smoke during pregnancy may increase the risk of postpartum depression in China

**DOI:** 10.18332/tid/87141

**Published:** 2018-04-24

**Authors:** Li Wang, Ke Fu, Xuri Li, Beihua Kong, Bin Zhang

**Affiliations:** 1 Liaocheng People’s Hospital, Medical College of Liaocheng University, Liaocheng, China; 2 Department of Gynecology and Obstetrics, Liaocheng NO.4 People’s Hospital, Liaocheng, China; 3 Department of Gynecology and Obstetrics, Qingdao Hiser Medical Group, Qingdao, China; 4 Shandong University, Qilu Hospital, Jinan, China; 5 Liaocheng People’s Hospital, Liaocheng, China

**Keywords:** third-hand smoke, pregnancy, postpartum depression

## Abstract

**INTRODUCTION:**

Our aim was to investigate the association between third-hand smoke exposure (THS) during pregnancy and postpartum depression (PPD) among Chinese women.

**METHODS:**

A total of 973 participants that completed the questionnaire and reviews were included in this cross-sectional study. The THS exposure was assessed based on a questionnaire with key questions from the Global Adult Tobacco Survey (2nd edition) while postpartum depression status was assessed using the Edinburgh Postnatal Depression Scale. Logistic regression models were used to estimate the association between THS exposure during pregnancy and risk of PPD, after adjustment for potential confounders.

**RESULTS:**

The prevalence of postpartum depression among all participants was 17.8%. Of the 973 participants, 725 (74.5%) were exposed to THS during pregnancy while 248 (25.5%) were not. Compared with those who were never exposed to passive smoking, puerperal women who were exposed to THS were at higher risk of PPD (OR=1.71, 95% CI: 1.12–2.60) in the logistic regression model.

**CONCLUSIONS:**

Our study suggests that exposure to third-hand smoke may be a risk factor for postpartum depression among Chinese women. Future preventive interventions should include strategies that target the puerperal women who are exposed to THS during pregnancy. Tobacco control measures that are effective in reducing the prevalence of smoking may have little effect in reducing women’s exposure to THS during pregnancy, especially in private places that urgently need other public health strategies.

## INTRODUCTION

Postpartum depression (PPD), also known as postnatal depression, is a serious mood disorder occurring after delivery that can affect new mothers for up to 1 year post-delivery^1,2^. The prevalence of PPD varies from country to country, a literature review of 143 research studies conducted in 40 countries demonstrated that the prevalence of PPD was estimated to vary from almost 0.5% to over 61.5%^3^. It is a significant mental health problem that disrupts a woman’s life, and has adverse effects on mothers, children and families. PPD could increase the risk of self-harm, suicidal thoughts and infanticide^4,5^. It can affect maternal-infant interaction patterns, which in turn might contribute to a disturbed neurobiological development of her child^6^, including emotional, cognitive and behavioral problems and low social competence^7,8^.

Third-hand smoke (THS) has been the focus in recent discussions on another form of tobacco exposure - defined as residual tobacco smoke contaminants and byproducts on materials such as clothing after extinguishing a tobacco product^9,10^. THS can remain on indoor walls, fabrics and other typical household items, such as curtains, carpets, sofa upholstery, beds and chairs for more than 1.5 years^11,12^. Tobacco use, including active smoking and exposure to tobacco smoke, is one of the leading risk factors for premature mortality and disability from non-communicable diseases in China^13^. While smokers may protect family members against secondhand smoke (SHS) at home by smoking outside, their exhaled breaths, skin, hair and clothing may transfer smoke residues back into the home, exposing the family to THS^14-17^. Unlike SHS, THS routes of exposure are not limited to inhalation, but also to ingestion of THS-loaded dust and dermal contact with contaminated surfaces^18^. The nicotine exposure levels and tobacco-specific nitrosamines can be much higher from THS than from SHS in both children and adults^11^.

Several research studies using animal models indicated that THS exposure might affect lung development and contribute to lung diseases, cardiovascular disease, poor wound healing and hyperactivity^12,19^. Some studies have suggested that cigarette use in active smokers may affect chronic depression, but their findings did not mention specifically pregnant women^20-22^. A few researchers have studied the relationship between SHS and risk of depression during pregnancy or PPD^23-25^. However, being a hidden source of tobacco smoke, THS exposure and its harms have been overlooked in many other studies; to our knowledge there are no studies that have examined the association between THS and PPD. Therefore, we investigated the prevalence of pregnant women to THS exposure and the associations with postpartum depression in never smokers.

## METHODS

### Participants in study

This cross-sectional study was carried out in Liaocheng People’s Hospital, Qilu Hospital, Qingdao Hiser Medical Group and Liaocheng No.4 People’s Hospital, located in Shandong Province, in the east of China. There are more than 20 000 deliveries in these hospitals each year.

A total of 4215 deliveries occurred during February, May, August and November in 2014. We invited 4000 women to come to the hospital to take part in a postpartum checkup within 2–8 weeks after delivery, but not all women who gave birth in the hospital came back to the same hospital for their checkup. A total of 1877 women returned to the hospital and completed our questionnaire via a face-to-face interview with a professional psychiatrist. In the current analysis, we excluded 904 (48.2%) participants with missing information regarding THS exposure or PPD. Finally, 973 participants were included in our study.

The study protocol was approved by the ethics committee of the Liaocheng People’s Hospital, Qilu Hospital, Qingdao Hiser Medical Group and Liaocheng No.4 People’s Hospital. All participants provided a written informed consent form.

### Measurements of exposure variable

We used the Edinburgh Postnatal Depression Scale (EPDS) that has a 10-item self-reported screening tool for PPD^26^ to assess postpartum depression in our study. Respondents were asked to rate the intensity of their depressive symptoms in the past 7 days using 4-point Likert-type scales with each category of response ranging from 0 (i.e. not at all) to 3 (i.e. quite often) with a total possible score ranging from 0 to 30. Higher scores often indicated worse postpartum depression.

A standardized questionnaire adapted from the Global Tobacco Adult Survey was sent to these women. To describe their smoking status during pregnancy, mothers were provided with two options, including ‘never smoking’ and ‘ever smoking’. They were also asked the number of days that they smoked in the past 30 days before pregnancy. Those choosing ‘never smoking’ and reporting no smoking in the past 30 days before pregnancy were defined as never smokers. The main exposure variable in this study was prenatal THS exposure. THS exposure at home was assessed by ‘During your most recent pregnancy, how many days did you smell cigarette smoke at home when nobody was smoking at home, but someone might have smoked earlier?’, THS exposure outside the home was assessed by ‘During your recent pregnancy, how many days did you smell cigarette smoke outside your home such as in an office room or in other places when nobody was smoking, but someone might have smoked earlier?’. The response options for each exposure, 0 to 7 days, were categorized into ‘0 day/week’, ‘1–4 days/week’, and ‘5–7 days/week’^27^. Women who replied ‘none’ or ‘0 day/week’ to this question were considered as ‘unexposed’ and women who replied ‘1–4 days/week’ or ‘5–7 days/week’ were considered as ‘exposed’ to THS during pregnancy. Other socio-demographic characteristics including age, household registration, education level, parity and medical insurance were also recorded.

### Statistical analysis

SPSS 19.0 and Prism graph were used for analysis, prevalence of exposure to the tobacco smoke, THS at home and outside the home during pregnancy were calculated. Continuous variables were summarized as mean ± standard deviation (SD). Student t-tests were used to examine differences in means for continuous variables among participants with PPD and those without PPD. Categorical variables were presented as proportion (%) and compared using Chi-squared tests. All statistically significant variables (p<0.05) were retained in the final logistic model. Logistic regression analyses were performed to estimate odds ratios (ORs) and 95% confidence intervals (95% CIs) of the association between different forms of THS and risk of PPD. All the statistical tests were two-tailed, and the cutoff of significant level was defined as p<0.05.

## RESULTS

A total of 973 women were analyzed after excluding those with missing information. Of the 973 puerperal women, 17.8% (173) had PPD and the prevalence of THS exposure during pregnancy was 74.5% (725). The mean age of the participants was 28.6 ± 5.44 years.

Table 1 shows basic characteristics according to PPD of the women participating in the study. Primipara with maternal smoking, exposure to THS during pregnancy, mothers with lower educational level and delivery by cesarean were more likely to develop PPD (p<0.05), and so does caregiver (mother-in-law) and artificial feeding after delivery (p<0.01).

**Table 1:** Characteristics of participants according to postpartum depression

*Characteristics*	*Depressed (N=173)*	*Not Depressed (N=800)*	*p*
**Age**	28.7±4.81	28.5±5.93	
**Occupation**			
House wife (%)	67 (38.7%)	266 (33.3%)	0.17
Employed (%)	106 (61.3%)	534 (66.8%)	
**Highest education**			
Junior high school or below (%)	25 (11.5%)	192 (24.0%)	0.01
High school or above (%)	148 (85.5%)	608 (80.4%)	
**Household registration**			
Native (%)	72 (41.6%)	314 (44.2%)	0.54
Immigrant (%)	101 (58.4%)	396 (55.8%)	
**Income (¥¥/person/month)**			
≤10 000 (%)	103 (59.5%)	473 (59.1%)	0.92
>10 000 (%)	70 (40.5%)	327 (40.9%)	
**Medical recipient**			
No (%)	130 (75.1%)	613 (76.6%)	0.67
Yes (%)	43 (40.5%)	187 (23.4%)	
**Smoking status**			
Never smokers (%)	129 (74.6%)	740 (92.5%)	0.00
Ever smokers (%)	44 (25.4%)	60 (7.50%)	
**Exposure to thirdhand smoke**			
No (%)	31 (17.91%)	217 (27.13%)	0.01
Yes (%)	142 (82.08%)	583 (72.87%)	
**Parity**			
Primiparous (%)	96 (55.5%)	370 (46.3%)	0.02
Parous (%)	77 (44.5%)	430 (53.8%)	
**Delivery mode**			
Vaginal delivery (%)	92 (53.2%)	574 (71.4%)	0.00
Caesarean section (%)	81 (46.8%)	226 (28.6%)	
**Infant gender**			
Girl (%)	78 (45.1%)	377 (46.9%)	0.67
Boy (%)	95 (54.9%)	423 (53.1%)	
**Caregiver of puerperal women**			
Mother-in-law (%)	75 (43.4%)	231 (28.9%)	0.00*
Mother (%)	30 (17.3%)	236 (29.5%)	
Maternity matron (%)	68 (39.3%)	333 (41.6%)	
**Feeding pattern**			
Brest feeding (%)	39 (22.5%)	291 (36.4%)	0.00*
Mixed feeding (%)	66 (38.2%)	377 (47.1%)	
Artificial feeding (%)	68 (39.9%)	132 (16.5%)	

Categorical variables are presented as number (%) and continuous variables are summarized as mean ± SD.

* Fisher test

Overall, 74.5% of pregnant women were exposed to THS at home or outside the home (Table 2). THS exposure at home, referring to smelling residual cigarette smoke, was reported by 47.5% of pregnant women, including 23.1% for 1–4 days and 24.4% for 5–7 days/week. THS exposure outside the home was reported by 58.5% of the pregnant women, including 27.5% for 1–4 days and 31.0% for 5–7 days/week. Of the PPD women, 82.1% were exposed to THS during their pregnancy, including 35.8% for 1–4 days and 46.2% for 5–7 days. Of all the 800 not-depressed women, 72.9% reported THS exposure during pregnancy, including 27.1% for 1–4 days and 32.9% for 5–7 days.

**Table 2:** Prevalence of THS exposure anywhere in pregnant women and PPD (N=973)

*Exposure (day/week)*	*THS*			*Depression status*	
	*At home (%)*	*Outside home (%)*	*n*	*Depressed (%)*	*Not Depressed (%)*
0	–	–	248	17.9	22.3
1–4	23.1	27.6	325	35.8	35.4
5–7	24.4	30.9	400	46.2	37.5
All	47.5	58.5	973	82.1	72.9

Compared with those who were never exposed to THS during pregnancy, women who were exposed to a THS environment during pregnancy were at a higher risk of PPD (OR=1.70, 95% CI: 1.12–2.60) in the crude logistic regression model. When different exposure and several socio-demographic characteristics were adjusted, the association for THS exposure during pregnancy and PPD remained significant, with COR (95% CI) of 1.65 (1.03–2.63) and AOR (95% CI) of 1.46 (1.01–2.35) for 1–4 days. COR (95% CI) was 1.75 (1.12–2.74) and AOR (95% CI) was 1.51 (1.02–2.46) for 5–7 days (p for trend<0.001) (Table 3). In both crude and adjusted models, pregnant women exposed to more THS had higher odds of developing PPD, compared with those unexposed to THS.

**Table 3:** Odds ratios (95% CI) of postpartum depression according to exposure to THS

*Exposure (day/week)*	*PPD n (%)*	*COR (95% CI)*	*AOR (95% CI)*	*p for trend*
0	31 (17.9%)	1.0 (Ref)	1.0 (Ref)	
1–4	62 (35.8%)	1.65 (1.03–2.63)	1.46 (1.01–2.35)	0.01
5–7	80 (46.2%)	1.75 (1.12–2.74)	1.51 (1.02–2.46)	

COR=crude odds ratio, AOR=adjusted odds ratio, CI=confidence interval, THS=third-hand smoke. Different exposure sources were adjusted mutually and for age, highest education, smoking status, parity, delivery mode, caregiver of puerperal women, and feeding pattern.

## DISCUSSION

This study is the first to include THS as a separate variable in involuntary tobacco smoke exposure in pregnancy and its association with postpartum depression in women. Our study shows that pregnant women who were exposed to THS had a nearly twofold increased risk of PPD compared with those without exposure. When the analysis was adjusted for confounding, the exposure outcome relationship was weakened but remained statistically significant.

To our knowledge, some epidemiological studies have shown that smoking and depression often go together^28^ and explored the effect of passive smoking on the depression syndromes. At present, there is some evidence for a relation between SHS exposure and depressive symptoms or poor mental health^29-33^. While the adverse health effects of smoking and secondhand cigarette smoke exposure are well known^34,35^, third-hand smoke (THS) has only recently emerged as a public health concern^9^. The concept of THS as a distinct entity that poses health risks for people has been developed only recently. Researches estimate that a single cigarette smoked on one day in a given environment can expose many people to the smoke’s toxic compounds for days and even months^36^. One study in California (USA) published in 2011^37^ showed that in homes previously inhabited by smokers that had been vacant even for two months, nicotine was still detected in the air, dust and surfaces of the furniture in the bedroom and living room for a month after the new non-smoking residents had moved in. Even in places where smoking bans are strictly enforced, such as neonatal intensive care units in hospitals, THS can be found by measuring in infants’ urine^15^ the concentrations of (4-(methylnitrosamino)-1-(3-pyridyl)-1-butanol) (NNAL)/cotinine. Ramirez et al.^38^ by analyzing nicotine and nitrosamines/TSNAs in house samples found that the calculated cancer risk for children (1 to 6 years old) increased. In addition, low level prenatal exposure to tobacco smoke correlates with cognitive deficits in infants, suggesting that some compounds in tobacco smoke may be neurotoxic^39^. Ramirez et al. also estimated the risk of cancer from exposure to carcinogens in THS.

Since pregnant women typically spend more time indoors, they are often in close contact with surfaces and dust. Furthermore, pregnant women are more sensitive than others to pollutants for several reasons, including increased respiration, abdominal size, disabled in action, changes of immunologic systems, and changes of metabolic capacity. Thus, even low doses of THS constituents may represent a long-term health hazard to them and their babies. Although these previous studies suggest that THS is a potential health threat to infants and young children who are in smokers’ homes, virtually nothing is known about the exposure to THS components, since there are few studies on its effects, and there are still few studies on the effects of THS on pregnant women and their mental health.

To note, so far only two studies explored this relation among the pregnant population (one for antenatal depressive symptoms and the other for postpartum depressive symptoms), and found that SHS exposure was positively associated with depressive symptoms^23,24^.

This study builds on previous literature on passive smoking exposure in homes or work-places to evaluate setting-specific association and dose-response observed between THS and PPD to make the prevention more accurate. Over 70% women were exposed to THS during their pregnancy and more than 17% had PPD. The prevalence might have been underestimated as THS exposure at weak and unnoticeable levels could not be captured by our question. However, the presence and persistence of toxic compounds with harmful health effects mean that the danger of THS cannot be ignored. Half of the nicotine deposited on clothing and dissolved by sweat can penetrate the body through the skin and be absorbed^40^. China has published comprehensive tobacco control measures these years and the smoke-free areas were greatly extended to most indoor public places, all indoor restaurants and workplaces, and some outdoor places in 2016, and progressively extended to cover more public places afterwards. Still, a substantial proportion of pregnant women were exposed to tobacco smoke in places where no one was smoking at that time. Our findings provide strong evidence to support comprehensive smoke-free house and workplace policies, which should ban smoking in not only shared areas but also in private homes. These findings also support that future studies could investigate whether infants’ mental disorders were linked to THS exposure at home and the number of sources of tobacco smoke exposure.

The strengths of our study include a large sample size and abundant potential confounders benefit by the strict postpartum follow-up system in China. First, it is a pilot research project that assesses the relationship between THS and PPD. Second, we contributed to the literature by exploring the dose-response relations between both frequency of THS exposure and PPD. Furthermore, interviews were performed within a hospital by professional psychologists and gynecologists, to make the data more reliable. However, potential limitations also need to be considered. First, our study was cross-sectional; therefore, although we demonstrated that there was a higher risk of PPD in puerperal women who were exposed to THS during pregnancy, causal and temporal relationships could not be inferred. Second, we did this analysis in Shandong Province located in east China, so our data may not be representative of pregnant women across all China. Third, although the strength of this study is the source of THS exposure, it is limited by self-report and has no biochemical measurements, such as that of cotinine. Self-reported exposure to THS allowed the prevalence, frequency and duration of THS exposure to be assessed, but not the ‘quality’ of THS exposure (e.g. nicotine concentration in the air). In addition, depressive symptoms were also based on self-report, and thus were subject to recall bias and reporting bias. We only utilized EPDS to assess PPD under the professional psychologists’ help without confirming the diagnosis with DSM-IV-TR (Diagnostic and Statistical Manual of Mental Disorders, Fourth Edition, Text Revision). The self-reported method yields significantly higher estimates of PPD than the clinical psychiatric interview-based method^3^. Additionally, it has been reported that some women with PPD had already shown depressive symptoms during pregnancy^41^. Unfortunately, we do not know if these women were depressed during their pregnancy, which may be implicated in the development of PPD^42^, such a recall bias might have occurred.

## CONCLUSIONS

Our study suggests that exposed to THS is a risk factor for PPD among Chinese women. These findings enhance our understanding of risk factors for PPD and emphasize the need to address the risk of PPD in Chinese puerperal women.

## CONFLICTS OF INTEREST

Authors have completed and submitted the ICMJE Form for Disclosure of Potential Conflicts of Interest and none was reported.

## References

[1] Miller LJ (2002). Postpartum depression. JAMA.

[2] Roehr B (2013). American Psychiatric Association explains DSM-5. BMJ.

[3] Halbreich U, Karkun S (2006). Cross-cultural and social diversity of prevalence of postpartum depression and depressive symptoms. J Affect Disord.

[4] Friedman SH, Resnick PJ (2009). Postpartum depression: an update. Womens Health (Lond).

[5] Wisner KL, Sit DK, McShea MC (2013). Onset timing, thoughts of self-harm, and diagnoses in postpartum women with screen-positive depression findings. JAMA PSYCHIAT.

[6] Brummelte S, Galea LA (2016). Postpartum depression: Etiology, treatment and consequences for maternal care. HORM BEHAV.

[7] Luoma I, Tamminen T, Kaukonen P (2001). Longitudinal study of maternal depressive symptoms and child well-being. J Am Acad Child Adolesc Psychiatry.

[8] Beck CT (1998). The effects of postpartum depression on child development: a meta-analysis. Arch Psychiatr Nurs.

[9] Matt GE, Quintana PJ, Destaillats H (2011). Thirdhand tobacco smoke: emerging evidence and arguments for a multidisciplinary research agenda. Environ Health Perspect.

[10] Sleiman M, Logue JM, Luo W, Pankow J, Gundel L, Destaillats H (2014). Inhalable constituents of thirdhand tobacco smoke: chemical characterization and health impact considerations. Environ Sci Technol.

[11] Bahl V, Jacob PR, Havel C, Schick S, Talbot P (2014). Thirdhand cigarette smoke: factors affecting exposure and remediation. PLoS One.

[12] Rehan VK, Sakurai R, Torday JS (2011). Thirdhand smoke: a new dimension to the effects of cigarette smoke on the developing lung. Am J Physiol Lung Cell Mol Physiol.

[13] Yang G, Wang Y, Wu Y, Yang J, Wan X (2015). The road to effective tobacco control in China. Lancet.

[14] Invernizzi G, Ruprecht A, De Marco C, Paredi P, Boffi R (2007). Residual tobacco smoke: measurement of its washout time in the lung and of its contribution to environmental tobacco smoke. Tob Control.

[15] Northrup TF, Khan AM, Jacob PR (2016). Thirdhand smoke contamination in hospital settings: assessing exposure risk for vulnerable paediatric patients. Tob Control.

[16] Matt GE, Quintana PJ, Hovell MF (2004). Households contaminated by environmental tobacco smoke: sources of infant exposures. Tob Control.

[17] Matt GE, Quintana PJ, Hovell MF (2004). Households contaminated by environmental tobacco smoke: sources of infant exposures. Tob Control.

[18] Becquemin MH, Bertholon JF, Bentayeb M (2010). Third-hand smoking: indoor measurements of concentration and sizes of cigarette smoke particles after resuspension. Tob Control.

[19] Martins-Green M, Adhami N, Frankos M (2014). Cigarette smoke toxins deposited on surfaces: implications for human health. PLoS One.

[20] Dierker LC, Avenevoli S, Stolar M, Merikangas KR (2002). Smoking and depression: an examination of mechanisms of comorbidity. Am J Psychiatry.

[21] Kendler KS (1993). Smoking and Major Depression. Archives of General Psychiatry.

[22] Paperwalla KN, Levin TT, Weiner J, Saravay SM (2004). Smoking and depression. Med Clin North Am.

[23] Mbah AK, Salihu HM, Dagne G, Wilson RE, Bruder K (2013). Exposure to environmental tobacco smoke and risk of antenatal depression: application of latent variable modeling. Arch Womens Ment Health.

[24] Khan S, Arif AA, Laditka JN, Racine EF (2015). Prenatal exposure to secondhand smoke may increase the risk of postpartum depressive symptoms. J Public Health (Oxf).

[25] Tan S, Courtney LP, El-Mohandes AA (2011). Relationships between self-reported smoking, household environmental tobacco smoke exposure and depressive symptoms in a pregnant minority population. Matern Child Health J.

[26] Cox JL, Holden JM, Sagovsky R (1987). Detection of postnatal depression. Development of the 10-item Edinburgh Postnatal Depression Scale. Br J Psychiatry.

[27] Leung LT, Ho SY, Wang MP, Lam TH (2018). Secondhand Smoke From Multiple Sources, Thirdhand Smoke and Respiratory Symptoms in Hong Kong Adolescents. Nicotine Tob Res.

[28] Bot M, Vink JM, Willemsen G (2013). Exposure to secondhand smoke and depression and anxiety: a report from two studies in the Netherlands. J Psychosom Res.

[29] Nakata A, Takahashi M, Ikeda T, Hojou M, Nigam JA, Swanson NG (2008). Active and passive smoking and depression among Japanese workers. Prev Med.

[30] Bandiera FC, Arheart KL, Caban-Martinez AJ (2010). Secondhand smoke exposure and depressive symptoms. Psychosom Med.

[31] Bandiera FC, Richardson AK, Lee DJ, He JP, Merikangas KR (2011). Secondhand smoke exposure and mental health among children and adolescents. Arch Pediatr Adolesc Med.

[32] Lee KJ (2014). Current smoking and secondhand smoke exposure and depression among Korean adolescents: analysis of a national cross-sectional survey. BMJ Open.

[33] Hamer M, Stamatakis E, Batty GD (2010). Objectively assessed secondhand smoke exposure and mental health in adults: cross-sectional and prospective evidence from the Scottish Health Survey. Arch Gen Psychiatry.

[34] Centers for Disease Control and Prevention Smoking and Tobacco Use.

[35] U.S. Department of Health and Human Services (2014). The Health Consequences of Smoking-50 Years of Progress: A Report of the Surgeon General. Reports of the Surgeon General.

[36] Escoffery C, Bundy L, Carvalho M (2013). Third-hand smoke as a potential intervention message for promoting smoke-free homes in low-income communities. Health Educ Res.

[37] Matt GE, Quintana PJ, Zakarian JM (2011). When smokers move out and non-smokers move In: residential thirdhand smoke pollution and exposure. Tob Control.

[38] Ramirez N, Ozel MZ, Lewis AC, Marcé RM, Borrull F, Hamilton JF (2014). Exposure to nitrosamines in thirdhand tobacco smoke increases cancer risk in non-smokers. Environ Int.

[39] Yolton K, Khoury J, Xu Y (2009). Low-level prenatal exposure to nicotine and infant neurobehavior. Neurotoxicol Teratol.

[40] Hammer TR, Fischer K, Mueller M, Hoefer D (2011). Effects of cigarette smoke residues from textiles on fibroblasts, neurocytes and zebrafish embryos and nicotine permeation through human skin. Int J Hyg Environ Health.

[41] Faisal-Cury A, Menezes PR (2012). Antenatal depression strongly predicts postnatal depression in primary health care. Rev Bras Psiquiatr.

[42] Clout D, Brown R (2015). Sociodemographic, pregnancy, obstetric, and postnatal predictors of postpartum stress, anxiety and depression in new mothers. J Affect Disord.

